# Validation of the cancer fatigue scale (CFS) in a UK population

**DOI:** 10.1186/s12885-025-14829-y

**Published:** 2025-08-28

**Authors:** C. Robinson, G. Feder, AL Huntley, J. Ramsay, D. Varsani, D. Gangahar, M. Kröz

**Affiliations:** 1 Centre for Evaluation and Methods, Pragmatic Clinical Trials Unit, Queen Mary University of London, London, UK; 2 Centre for Academic Primary Care, Bristol Medical School, University of Bristol, Bristol, UK; 3 Research Department, Klinik Arlesheim, Arlesheim, Switzerland; 4 Institute for Integrative Medicine, University Witten/Herdecke, Witten, Germany

## Abstract

**Background:**

Cancer-related fatigue is one of the most common symptoms in patients with cancer. Fatigue reduces health-related quality of life. The Cancer Fatigue Scale (CFS) is a brief instrument that captures the multidimensional nature of fatigue with robust validity. The original scale was developed/validated with Japanese cancer patients. It was our aim to report the psychometric properties of an English translation of the CFS.

**Methods:**

This cross-sectional study was conducted between February-October 2008 in London, UK. Three groups were recruited: cancer patients (index group), diabetic patients (chronic disease control group), and a healthy control group. All participants completed a questionnaire on recruitment and a subset were retested at 2–8 weeks. Reliability was assessed with analysis of internal consistency (Cronbach’s alpha), homogeneity (item-total correlations) and test-retest reliability. Construct validity was assessed with inter-subscale correlations. To assess convergent validity, the correlations between the CFS and FNS, EORTC − QLQ C30, and HADS were examined with Spearman Rank correlation coefficients. The discriminant validity was determined by the *known group* method.

**Results:**

160 participants were recruited: 60 cancer and 50 diabetes patients, and 50 healthy patients. Cronbach’s alphas for the total scale and all three subscales (range 0.83,0.94) were all above the 0.70 criterion suggested for group comparisons thus the CFS showed excellent internal consistency. Homogeneity measures all fulfilled the 0.20 criterion (range 0.53–0.84). Combining the three groups, all test-retest correlations for the CFS and its subscales were above 0.7. Physical and affective subscales showed 100% scaling success. Both scores and sub-scores for the CFS correlated positively (*p* < 0.001) with FNS and HADS depression and anxiety subscales. The CFS correlated positively (*p* < 0.001) with all but one item of the EORTC-QLQ C30.

**Conclusions:**

This evaluation of the measurement properties of the CFS-English shows that it is a reliable and valid tool for assessing key dimensions of cancer-related fatigue.

## Background

The health-related quality of life (HRQL) of cancer patients is diminished by a variety of factors, including pain, nausea and fatigue [1–3] Cancer-related fatigue (CRF), one of the most common symptoms in patients with cancer is “…. a distressing, persistent sense of physical, emotional and/or cognitive tiredness or exhaustion related to cancer or cancer treatment that is not proportional to recent activity and interferes with usual functioning.” [4] Often fatigue is one of the first presenting symptoms [5, 6]. In a study of newly diagnosed breast cancer and prostate cancer patients, the prevalence of severe fatigue was 15% and 16%, respectively [7]. Following oncological treatment, fatigue often increases. In cancer patients receiving active treatments such as cytotoxic chemotherapy, radiation therapy, bone marrow transplantation, or treatment with biological response modifiers, such fatigue is almost universal [4]. Chemotherapy-related CRF peaks in the days after chemotherapy, whereas radiation therapy-related CRF gradually accumulates over the course of treatment [8]. The CRF associated with both forms of treatment gradually declines over time, but can be long lasting [8]. Even after treatment, when the cancer is in remission, many patients continue to experience persistent fatigue. One study of breast cancer survivors found 34% of study participants reported significant fatigue at 5–10 years after diagnosis [9]. 

The aetiology of cancer fatigue is not well understood but is almost certainly multi-factorial in origin [10]. Pathophysiological factors (such as anaemia and cachexia), the effects of chemotherapy or radiotherapy on inflammatory cytokines, immune function, neuroendocrine, or circadian function [11–14], as well as psychological co-morbidity (such as depression, anxiety) and sleep disturbance [15] may all play a part. The effects of these various factors vary by cancer, disease stage, and treatment, and by the individual patient [16]. 

Being fatigued has a profound negative effect on the HRQL of people with cancer, as it can interfere with physical, psychological, social and spiritual dimensions of well-being [17]. In a group of heterogenous cancer patients, it was found that fatigue adversely affected the daily activities of patients, with 32% rating this as significant and 39% as moderate [18]. In one study of cancer patients, it was found that fatigue was the most commonly reported symptom and the most likely to interfere with everyday activities [19]. Yet, symptoms such as pain, nausea and depression are relatively well-managed with medication, whereas treatments for fatigue are less effective [20]. The National Comprehensive Cancer Network highlights that cancer-related fatigue has been under-reported, under-diagnosed and under-treated [4]. 

More effective management of fatigue could significantly reduce the impact of cancer on patients’ lives. This requires accurate identification of fatigue [21]. In systematic reviews of CRF scales, four scales are recommended [22, 23]. Such brief scales may be useful for screening purposes in clinical settings. However, they do not address the multidimensional nature of cancer-related fatigue (CRF), including cognitive and emotional components which are associated with chemotherapy [24]. Beside classic physical fatigue, reflecting a lack of vitality and altered muscle functions, the cognitive dimension of fatigue has an important impact in patients treated with chemotherapy including long-term memory disturbances, word finding difficulties and slow executive functions. Finally, patients with CRF often have a flattening of emotion and loss of interest in daily activities. Evaluation of treatments for CRF needs to include measurements of these components of fatigue. Yet we must avoid burdensome questionnaires, particularly with a fatigued population.

The Cancer Fatigue Scale (CFS) is an instrument that captures the multidimensional nature of fatigue with robust validity and is relatively brief [25]. The CFS is a 15-item self-reporting scale, assessing three components of CRF: physical, cognitive and affective. It was developed and validated in a heterogeneous Japanese cancer patient population, and was shown to have convergent validity, internal consistency and test-retest reliability [25]. Moreover, the instrument can easily be completed in two minutes. The instrument has been translated and validated in other languages: Chinese [26], German (CFS-D) [27] Persian [28], Greek [29] and Turkish [30]. Here we report the psychometric properties (reliability and validity) of an English translation of the CFS, a translation produced by the developers of the original Japanese version (see Appendix 1).

## Methods

The study was approved by the local office of the UK NHS Research Ethics Committee in accordance with the Declaration of Helsinki as a statement of ethical principles for medical research involving human subjects, including research on identifiable human material and data.

### Study design and participants

This multi-centre cross-sectional study was carried out between February and October 2008 in London, UK. Three participant groups were recruited: cancer patients (the index group), diabetic patients (a chronic disease control group), and people with no long-term health condition (a healthy control group).

The index patients were recruited from specialist oncology clinics. Eligibility criteria aged 18 years or older; ambulatory; tissue diagnosis of a malignancy requiring surgical intervention or chemotherapy, radiotherapy, hormonal, bisphosphonate or monoclonal antibody therapy; no surgical, chemotherapeutic or radiotherapeutic treatment within the last two weeks; a Karnofsky Index score greater than 40; unimpaired cognitive status; and a good understanding of English [31]. Participants in the two control groups were matched with the cancer patients according to age (± 2.5 years) and sex. Participants in the chronic disease control group were either recruited from a diabetes clinic at a teaching hospital or from the diabetes disease register of a large general practice. Eligibility criteria: a diagnosis of type 1 or type 2 diabetes mellitus without a malignancy; unimpaired cognitive status; and a good understanding of English. Participants in the healthy control group were recruited from the patient register of the same general practice. Eligibility criteria: no diagnosis of malignancy, diabetes or any other chronic medical or psychiatric condition. In all three patient groups, participants were excluded from the study if they were unable to self-complete a questionnaire, or felt too unwell to take part.

### Data collection

Patients attending the oncology and diabetes clinics were approached by a researcher. If a patient expressed an interest in participating, the researcher checked that inclusion criteria were met and obtained written consent. Freely-given, informed consent to participate in the study was obtained from all participants. To enable the recruitment of eligible control participants, the general practice register was searched for patients that were age/sex-matched with the index cancer patients. For each cancer patient, four matched people with diabetes and four matched healthy people were selected. Details of the study were then posted to these patients. If there was no response after two weeks, one reminder was sent. For participants recruited by post, consent was implied by completion of the questionnaire.

All participants who completed a questionnaire at the time of recruitment (Time 1) were contacted by post and asked to complete a second questionnaire 2–8 weeks after the initial assessment (Time 2). At Time 2, all participants were asked an additional question about whether there was any difference in their health since they had first completed the first questionnaire. The cancer patients were asked to confirm that they had not received chemotherapy or radiotherapy in the intervening period. If such treatment had occurred, the patients were instructed to tick the relevant box on the opt-out sheet and not to complete the second questionnaire.

### Assessment instruments

The Cancer Fatigue Scale (CFS) is a self-report 15-item scale measuring current experience of fatigue in cancer patients. It comprises three subscales: physical fatigue (7-items), cognitive fatigue (4-items) and affective fatigue (4-items), scored from 1 (not at all) to 5 (very much) [25]. 

The Fatigue Numerical Scale (FNS) is a visual analogue scale measuring general fatigue on the day of completion where a score of 0 indicates no fatigue and a score of 100 indicates extreme fatigue [32, 33]. 

The European Organization for Research and Treatment of Cancer Quality of Life Core Questionnaire (EORTC QLQ-C30 version 3.0) is a 30-item scale specifically designed to assess the quality of life of cancer patients (primarily in the past week) [34]. There are five functional scales: physical (5-items), role (2-items), emotional (4-items), cognitive (2-items), and social (2-items). Additionally, there are three symptom scales: fatigue (3 items), nausea/vomiting (2-items), and pain (2-items). Five single items also measure other symptoms commonly reported by cancer patients (dyspnea, appetite loss, insomnia, constipation, and diarrhoea). Two items measure global health status, and one item measures the perceived financial impact of the disease and treatment. Most of the items are scored on a 4-point Likert scale, the exception being the two global status items which are rated on a scale of 1 to 7. The EORTC QLQ-C30 has been widely used and validated in diverse health care settings [35, 36]. 

The Hospital Anxiety and Depression Scale (HADS) is a widely used 14-item questionnaire measuring anxiety (7-items) and depression (7-items) in the last week [36]. Each item is scored on a 4-point Likert scale. High scores on either subscale (range 0–21) are suggestive of significant psychological concerns. The reliability and validity of the HADS has been established in numerous studies [37, 38]. 

### Sample size

The capacity for the CFS to discriminate between people with cancer and healthy people is an important test of its validity. To have an 80% power to detect a standardised mean difference of 0.75 (consistent with the validation of the German CFS), we needed 25 participants/group. As our sample of healthy people were recruited via general practice lists, not − as in the German study − from professionals employed in a research institution, the differences with the cancer or diabetes group was likely to be smaller. With 50 participants/group (150 total) we had 80% power to detect a 0.55 standardised mean difference between the patients with cancer and healthy controls.

### Analyses

All analyses were carried out in Stata 10.1.

### Demographic characteristics

Frequencies and median/inter-quartile range scores were used to describe the demographic characteristics of the three patient groups at Times 1 and 2.

### Acceptability

Acceptability of the CFS was evaluated through an examination of the proportion of missing data for each item and the distribution of response categories across all patient groups.

### Principal components analysis

To examine if the three-factor solution observed in the original validation of the CFS could be replicated, a scree plot analysis was conducted with data from all three patient groups. Next, a principal components analysis with varimax rotation was conducted to examine the structure of the CFS [39]. Only items with factor loadings greater or equal to 0.40 were considered eligible for inclusion on a subscale. Where an item loaded on more than one factor, the item was allocated to the subscale with the higher item loading if the difference was at least 0.15; for smaller differences, the item was allocated to the factor deemed most appropriate in terms of its conceptual meaning [39]. 

### Reliability, scaling assumptions, and construct validity

The reliability of the CFS was assessed using standard psychometric techniques, including an analysis of internal consistency (Cronbach’s alpha), homogeneity (item-total correlations) and test-retest reliability [40]. Item convergent and discriminant correlations were used to test the scaling assumptions of the CFS; each item should be more correlated to its own subscale (item convergent validity) than to the other subscales (item discriminant validity). Construct validity was assessed by examining the inter-subscale correlations; the correlations between the subscales should be modest if they are measuring different components of fatigue.

### Convergent and discriminant validity

To assess convergent validity, we examined the correlations between the CFS and the three comparison measures (FNS, EORTC − QLQ C30, and HADS) on the entire sample [25, 33, 34, 37]. Spearman Rank Correlation Coefficients were calculated to test the association between the measures. We predicted that high scores for cancer fatigue would correlate with higher scores on the FNS and HADS, and symptom subscales of the EORTC-QLQ C30 (i.e., positive correlations); and correlate with lower scores on the EORTC-QLQ C30 functioning subscales (i.e., negative correlations).

The discriminant validity of the CFS was determined by use of the “known group” method [40]. We predicted that the CFS scores would be significantly higher in the cancer patient group as compared with the healthy patients group. The Mann-Whitney U-test was used to test for between-group differences.

## Results

### Response rates and socio-demographic characteristics

A total of 160 participants were recruited: 60 patients with cancer, 50 patients with diabetes, and 50 healthy patients. Sixty-seven eligible patients with cancer were approached in clinics, and 60 agreed to take part (90%). Out of 65 patients with diabetes approached in clinics, 32 were recruited (49%); a further 18 participants responded positively to a postal questionnaire sent to 91 patients with diabetes in general practice (20%). Two hundred and one healthy patients were asked to take part, and 60 returned the questionnaires (30%). At follow-up, five patients with cancer were ineligible, as they had received treatment since completing the initial questionnaire and a further five had requested no further contact. We received follow-up questionnaires from just over half of the cancer and healthy patients (56% and 58%, respectively) compared with one-third (34%) of the patients with diabetes.

The demographic characteristics of the participants at times 1 and 2 are summarised in Table 1. In all three groups, the majority of the participants were female, and the median age was around 60 years.

**Table 1:** Demographic characteristics on patient sample at time 1 and time 2

	Group			
All participants at Time 1	Cancer *n* = 60	Diabetes *n* = 50	Healthy *n* = 50	All groups *N* = 160
Male, n (%)	15 (25%)	11 (22%)	12 (24%)	38 (24%)
Age in years, Median (IQR) Missing, n	60 (50, 72) 4	61 (50, 73) 0	59 (51, 69) 3	60 (50, 71) 7
Test re-test subset at Time 2	Cancer *n* = 28	Diabetes *n* = 17	Healthy *n* = 29	All groups *N* = 74
Male, n (%)	6 (21%)	2 (12%)	6 (21%)	14 (19%)
Age in years, Median (IQR) Missing, n	62 (53, 73) 0	63 (56, 74) 0	58 (50, 66) 1	60 (53, 71) 1

### Acceptability

Based on an examination of the proportion of missing data and the distribution of responses across patient groups, we concluded that the CFS is an acceptable measure. Across the three patient groups, 91% of participants answered all 15 items and a further 6% of patients answered all but one of the items. This level of response was similar across the three groups. There was no suggestion that any one item was difficult for the participants to answer, and this was reflected in the similar number of responses by all three patient groups for each of the three subscales. 94–96% of participants answered all items within the physical, affective, and cognitive subscales. Estimates of central tendency, dispersion and other features of score distributions are reported in Table 2. An almost full range of possible scores was exhibited by the cancer and diabetes groups, and there were no extreme ceiling or floor effects for these participants. As the scores indicate, most patients with cancer or diabetes reported medium levels of physical and affective fatigue, with low levels of cognitive fatigue. As expected, the range of scores in the healthy patient group was more restricted and only low levels of fatigue of any type were reported.

**Table 2:** Descriptive statistics and features of score distributions for CFS and subscales

	Group			
	Cancer *n* = 60	Diabetes *n* = 50	Healthy *n* = 50	All groups *N* = 160
Physical Subscale (range 0–28)				
N (Min, max)	56 (0,28)	46 (0, 28)	48 (0,19)	150 (0, 28)
Median (IQR)	11 (5, 16)	10 (3, 18)	4 (1, 8)	8 (2, 14)
Score of 0, n (%)	4 (7%)	6 (13%)	10 (21%)	20 (13%)
Score of 28, n (%)	1 (2%)	2 (4%)	0 (0%)	3 (2%)
Affective Subscale (range 0–16)				
N (Min, max)	59 (0, 15)	48 (0, 16)	47 (0, 16)	154 (0, 16)
Median (IQR)	8 (3, 10)	7 (2, 10)	4 (1,7)	6 (2, 9)
Score of 0, n (%)	1 (2%)	3 (6%)	7 (15%)	11 (7%)
Score of 16, n (%)	0 (0%)	1 (2%)	1 (2%)	2 (1%)
Cognitive Subscale (range 0–16)				
N (Min, max)	56 (0, 15)	49 (0, 14)	48 (0, 10)	153 (0, 15)
Median (IQR)	2 (4, 7)	3 (2, 7)	2 (0, 4)	3 (1,6)
Score of 0, n (%)	7 (13%)	9 (18%)	14 (29%)	30 (20%)
Score of 16, n (%)	0 (0%)	0 (0%)	0 (0%)	0 (0%)
Total score (range 0–60)				
N (Min, max)	54 (1, 57)	45 (0, 58)	47 (0, 37)	146 (0, 58)
Median (IQR)	22 (13, 29)	19 (9, 32)	11 (4, 19)	18 (7, 27)
Score of 0, n (%)	0 (0%)	2 (4%)	3 (6%)	5 (3%)
Score of 60, n (%)	0 (0%)	0 (0%)	0 (0%)	0 (0%)

### Principal components analysis

The results of the scree plot suggested a three-factor solution and we conducted a principal components analysis retaining three factors with a varimax rotation. The resulting three factors explained 64% of the common variance: factor 1 (physical fatigue) accounted for 52%, factor 2 (affective fatigue) for 7%, and factor 3 (cognitive fatigue) for 5% of the variance.

Examination of the factor loadings (Table 3) showed that three of the items (items 4, 9 and 12) loaded on two factors with less than a 0.15 difference. Item 9 (Do you feel fed up?) and item 12 (Do you feel reluctant?) loaded marginally higher on the physical subscale (0.4819 and 0.5250, respectively) as compared with the cognitive scale (0.4777 and 0.4446). This pattern of results is consistent with the original Japanese validation study where the two items ultimately were assigned to the physical subscale, a decision which the developers argue is further supported by similar factor loadings in two subsequent unpublished studies of the CFS [25]. 

**Table 3:** Factor loading pattern (after varimax rotation) of the CFS

Item number and content	Factor		
	Physical	Affective	Cognitive
3. Do you feel exhausted?	0.8041	0.2469	0.3092
2. Do you have the urge to lie down?	0.7825	0.3275	0.1339
1. Do you become tired easily?	0.7641	0.3368	0.2220
6. Does your body feel heavy and tired?	0.6579	0.3164	0.3885
15. Do you feel such fatigue that you don’t know what to do with yourself?	0.5913	0.2946	0.3702
12. Do you feel reluctant?	0.5250	0.3450	*0.4446*
9. Do you feel fed-up?	0.4819	0.3370	*0.4777*
14. Can you encourage yourself to do anything?	0.2671	0.7524	0.2284
8. Do you feel interest in anything?	0.3164	0.7280	0.1642
11. Can you concentrate on certain things?	0.2761	0.6781	0.1869
5. Do you feel energetic?	0.4244	0.6558	0.1561
13. Do you feel that your thinking has become slower?	0.2492	0.2122	0.7236
10. Do you feel you have become forgetful?	0.3066	0.2211	0.7035
7. Do you feel that you more often make errors while speaking?	0.2380	0.1565	0.6666
4. Do you feel you have become careless?	*0.5060*	0.0873	0.4872

Only items with factor loadings greater or equal to 0.40 were considered eligible for inclusion on a subscale

For this reason, we too allocated items 9 and 12 to the physical subscale. Item 4 (Do you feel you have become careless?) loaded more onto the physical scale (0.5060) than on the cognitive subscale (0.4872). This finding conflicted with the original Japanese study in which it loaded higher (by a difference of 0.13) onto the cognitive subscale than the physical scale. In conceptual terms, feelings of carelessness may relate to physical or cognitive fatigue. However, for reasons of consistency with the original subscale structure, we assigned this item to the cognitive subscale.

### Reliability and scaling assumptions

Internal consistency: the Cronbach’s alphas for the total scale and all three subscales (range 0.83 to 0.94) were all above the 0.70 criterion suggested for group comparisons and thus the CFS showed excellent internal consistency (Table 4) [41]. Deletion of item 4 (Do you feel you have become careless?) from the cognitive subscale was found to increase the alpha from 0.83 to 0.84.

**Table 4:** Reliability of the CFS

	Internal consistency and homogeneity				Test-retest	
CFS multi-item scales	Cronbach’s alpha coefficient	n	Range of alphas if individual items deleted	Range of item-total correlations	Test-retest correlations	n
Physical subscale	0.92	150	0.91–0.92	0.70–0.84	0.92	72
Affective subscale	0.87	154	0.83–0.85	−0.71 – −0.77	0.78	73
Cognitive subscale	0.83	153	0.75–0.84	0.53–0.75	0.82	71
Total scale	0.94	146			0.90	68

### Homogeneity

The homogeneity of the CFS was assessed by examining the item-total correlations (Table 4). Such correlations compute the correlation between an item and its own subscale once the item of interest has been eliminated from the calculation. Item-total correlations should be more than the criterion 0.20 [42]. All such correlations fulfilled this criterion (range 0.53 to 0.84).

### Test-retest reliability

Combining the three patient groups, all test-retest correlations for the CFS and its subscales were above 0.7 (Table 4) [43]. However, separate analyses by patient group (data not shown), showed that the responses of the diabetes group changed over time in relation to affective fatigue (*r* = 0.44) and cognitive fatigue (*r* = 0.60), as well as their total CFS scores (*r* = 0.69).

### Scaling assumptions

Using item convergent and discriminant correlation testing, the physical and affective subscales showed 100% scaling success. The cognitive subscale achieved only 75% scaling success; item 4 (Do you feel you have become careless?) correlating more highly with the physical subscale (*r* = 0.63) than its own subscale (*r* = 0.53).

### Inter-scale correlations

The inter-scale correlations provided some evidence of discriminant validity between the subscales. The correlation between the affective and cognitive subscales was modest (*r* = 0.51). Correlations between the physical subscale and the other subscales were slightly higher: affective (*r* = 0.71) and cognitive (*r* = 0.70).

### Convergent and discriminant validity

As predicted, scores for the CFS and its three subscales all correlated positively with the FNS, and the HADS subscales (*p* < 0.001) (Table 5). Similarly, the CFS (total and subscales) correlated positively with all but one of the symptom subscales/items of the EORTC-QLQ C30 (*p* < 0.001). The one exception was the correlation between the CFS cognitive subscale and the EORTC-QLQ C30 constipation symptom score (rho = 0.13, *p* = 0.11). The relationship between the CFS and the functioning subscales of the EORTC-QLQ C30 were all in the predicted negative direction (*p* < 0.001).

**Table 5:** Convergent validity: CFS comparisons with the FNS, EORTC-QLQ C30, HADS

CFS	FNS	HADS		EORTC-QLQ C30												
				Symptoms								Functioning				
		Anxiety	Depress -ion	Fatigue	Nausea/ vomiting	Pain	Dyspnea	Appetite loss	Insomnia	Constip -ation	Diarrhoea	Physical	Roles	Emotion -al	Cognitive	Social
Physica	0.59	0.62	0.74	0.80	0.31	0.54	0.53	0.38	0.52	0.20^b^	0.24^a^	−0.69	−0.68	−0.66	−0.66	−0.66
Affective	0.50	0.53	0.73	0.68	0.29	0.44	0.45	0.43	0.49	0.33	0.16^b^	−0.57	−0.62	−0.53	−0.60	−0.55
Cognitive	0.40	0.47	0.53	0.48	0.25^a^	0.20^b^	0.36	0.20	0.25	0.13^c^	0.21^b^	−0.45	−0.33	−0.52	−0.69	−0.33
Total	0.57	0.62	0.78	0.78	0.34	0.49	0.53	0.40	0.51	0.25^a^	0.23^a^	−0.66	−066	−0.65	−0.73	−0.61

^*^Number of participants completing: CFS Physical and comparison measures = 144–147; CFS Affective and comparison measures = 148–151; CFS Cognitive and comparison measures = 147–150; CFS Total scale and comparison measures = 138–143

^**^All comparisons significant at *p* < 0.001, except ^a^ *p* < 0.01, ^b^ *p* < 0.05, ^c^ n/s

In terms of discriminant validity (Table 6), we found that the patients with cancer or diabetes patients responded very similarly in their reporting of fatigue and the analysis did not reveal any statistically significant differences between the two groups, either in terms of the CFS total score or any of the three subscales. There were, however, significant differences in the levels of fatigue reported by the cancer patients and the healthy group. For all three subscales and the total CFS score, the cancer patients scored higher in comparison to the healthy participants.

**Table 6:** Discriminant validity

	Group			Mann Whitney test *p*-values	
	Cancer (median, IQR)	Diabetes (median, IQR)	Healthy (median, IQR)	Cancer versus Diabetes	Cancer versus Healthy
Physical subscale	11 (5, 16)	10 (3, 18)	4 (1, 8)	0.6326	0.0000
Affective subscale	8 (3, 10)	7 (2, 10)	4 (1, 7)	0.3946	0.0003
Cognitive subscale	2 (4, 7)	3 (2, 7)	2 (0, 4)	0.7762	0.0015
Total scale	22 (13, 29)	19 (9, 32)	11 (4, 19)	0.6029	0.0000

## Discussion

In this study, we have shown that the English translation of the CFS (CFS-E) has good psychometric properties, supporting the use of the CFS as a research tool to evaluate the key dimensions of fatigue experienced by cancer patients in English speaking populations.

The principal components analysis suggested a three-factor solution with loadings indicative of physical, affective and cognitive fatigue. These findings support those of the original Japanese version of the CFS [25, 44] as well as the versions translated into Chinese and German [26, 27]. However, while the general pattern of factor loadings was similar to that found in the original Japanese validation study, our findings differed in one important respect. In our study, item 4 (Do you feel you have become careless?) loaded slightly higher on the physical subscale, rather than the cognitive subscale. For conceptual consistency with the original version and the German version of the CFS, we still allocated this item to the cognitive scale. Nonetheless, it is clear that this item may be interpreted by some patients in terms of physical carelessness, and by others as cognitive carelessness. Indeed, in keeping with our findings, the researchers validating the Chinese translation of the CFS also found that item 4 loaded more highly on the physical domain as compared with the cognitive subscale [32]. It may be argued that changing the wording of this item might help to clarify to participants that this item refers to cognitive fatigue. For example: *Do you feel you have become careless in your thinking?* As indicated, the CFS-E meets the standard criteria for reliability, both in terms of its internal consistency and its test-retest reliability.

A particular strength of the CFS is its three dimensionality, capturing the complexity of cancer related fatigue. One of most burdensome disorders in patients undergoing chemotherapy is cognitive alteration, including memory dysfunction (forgetfulness), word finding difficulty, slower executive functioning and fine motor dysfunction, rigid problem solving [21, 45]. The CFS measures four of these problems [46, 47]. 

The CFS-E has good convergent validity. The correlation between the total CFS scale score and the FNS was reasonable, albeit slightly lower than that found in the original Japanese study [31] but higher than the CFS-D (*r* = 0.44).^33^ The correlations with the affective and cognitive subscales were of moderate size and thus illustrate that these aspects of fatigue also contribute to patients’ overall feelings of fatigue on a day-to-day basis. A similar pattern of correlations was found between the CFS-E and the EORTC-QLQ C30 fatigue symptom scale with strong correlations. The physical subscale of the CFS-E was the subscale most strongly correlated with EORTC-QLQ C30 fatigue, but the correlations with the affective and cognitive subscales, and the total CFS-E were also high, yet demonstrating the heterogeneity of cancer fatigue not detectable in the EORTC-QLQ C30 measure. As would be predicted, those items related to feelings and the complex construct of fatigue such as insomnia and pain were more pronounced than those less likely to be associated with fatigue, such as constipation and diarrhoea [34, 48]. 

Further evidence of the convergent validity of the CFS comes from its negative correlations with the functioning subscales of the EORTC-QLQ C30. The significant positive correlations between the CFS-E and the HADS anxiety and depression subscales likewise indicate that the CFS-E has good convergent validity. Anxiety and depression are known to be associated with fatigue – reflecting in the complex pathophysiology of CRF distress [37, 49]. 

The divergent validity of the CFS-E was established by our finding that the scores of healthy patients differed significantly from those of the cancer patients. This held true for the total CFS-E score and each of the three subscales. The scores of patients with cancer and diabetes did not differ. Diabetes patients often have sleep disturbances such as sleep apnoea or restless legs [50–52]. 

In terms of ease of completion, the authors of the Chinese version suggest that the CFS may be difficult to complete for some participants because the four items comprising the affective scale are positively worded whereas the other items are all negatively worded [26]. We did not find this to be a problem in our study, as more than 90% of participants responded to all 15 items of the CFS-E. Additionally, we did not find any differences relating to the mode of administration. We administered the CFS-E to patients in clinics and via post. Comparing the quality of the Time 1 data collected from cancer patients (who all completed the CFS in a clinic environment) and the healthy patients (who all received the CFS through the post), there is no evidence that one administration mode was better than the other. Thus, while the response rate was higher in the face-to-face situation, the CFS-E can be administered via the post and, potentially, via the internet. Similarly, the test-retest correlations shows that the mode of administration had a minimal effect on patients’ response choice, since more than half the sample at Time 1 completed the CFS-E in clinics and at Time 2 all patients were posted the questionnaire.

Strengths of our study include the comparison of three patient groups and rigorous testing of reliability and types of validity. Limitations include generalisability of our findings to patients with very poor function receiving palliative care. Although we specified a minimum Karnofsky index threshold of 40, all participants had a score greater than 60, making the three groups functionally comparable. A second limitation was that we did not systematically record details of cancer type or staging.

Further work to evaluate the performance of the CFS-E should focus on distinct cancer populations and treatment modalities. It may also have value to characterise and monitor fatigue in patients with rheumatological and neurological disorders, chronic fatigue syndrome or patients with primary sleep disorders [53, 54] A potential role in measuring treatment effects would need testing its sensitivity to change, as has been reported for the CFS-D, following multimodal CRF-treatments including sleep-education, psychoeducation, and mindfulness-orientated treatment (eurythmy) and art therapy[55], [56].

## Conclusion

This evaluation of the measurement properties of the CFS-E shows that it is a brief robust tool for assessing key dimensions of cancer-related fatigue. It is a reliable and valid instrument with the advantage of having a flexible mode of administration. It articulates those dimensions which other cancer fatigue measures do not, allowing evaluation of more specific responses to treatment in the context of clinical care and as a patient reported outcome measure for service evaluation and clinical trials. Its potential role as a research tool is wide ranging, although it needs further application to specific populations of patients with cancer and other chronic conditions.

## Data Availability

The datasets used and/or analysed during the current study are available from the corresponding author on reasonable request.

## Appendix 1

### The cancer fatigue scale

This questionnaire will ask you about any sense of fatigue you might be experiencing. For each question, please circle only one number that you think most aptly describes your current state. Try to answer on the basis of first impressions, without thinking deeply about each question.

**Table:**

Right now ….	No	A little	Somewhat	Considerably	Very much
1. Do you become tired easily?	1	2	3	4	5
2. Do you have the urge to lie down?	1	2	3	4	5
3. Do you feel exhausted?	1	2	3	4	5
4. Do you feel you have become careless?	1	2	3	4	5
5. Do you feel energetic?	5	4	3	2	1
6. Does your body feel heavy and tired?	1	2	3	4	5
7. Do you feel that you more often make errors while speaking?	1	2	3	4	5
8. Do you feel interest in anything?	5	4	3	2	1
9. Do you feel fed-up?	1	2	3	4	5
10. Do you feel you have become forgetful?	1	2	3	4	5
11. Can you concentrate on certain things?	5	4	3	2	1
12. Do you feel reluctant?	1	2	3	4	5
13. Do you feel that your thinking has become slower?	1	2	3	4	5
14. Can you encourage yourself to do anything?	5	4	3	2	1
15. Do you feel such fatigue that you don’t know what to do with yourself?	1	2	3	4	5
